# Ethnic Differences in Dementia Risk: A Systematic Review and Meta-Analysis

**DOI:** 10.3233/JAD-201209

**Published:** 2021-03-09

**Authors:** Suhail Ismail Shiekh, Sharon Louise Cadogan, Liang-Yu Lin, Rohini Mathur, Liam Smeeth, Charlotte Warren-Gash

**Affiliations:** Department of Non-Communicable Disease Epidemiology, London School of Hygiene & Tropical Medicine, Keppel Street, London, The United Kingdom

**Keywords:** Alzheimer’s disease, dementia, ethnic groups, incidence, prevalence

## Abstract

**Background:**

Globally around 50 million people have dementia. Risk factors for dementia such as hypertension and diabetes are more common in Black, Asian, and other ethnic minorities. There are also marked ethnic inequalities in care seeking, likelihood of diagnosis, and uptake of treatments for dementia. Nevertheless, ethnic differences in dementia incidence and prevalence remain under-explored.

**Objective:**

To examine published peer-reviewed observational studies comparing age-specific or age-adjusted incidence or prevalence rates of dementia between at least two ethnic groups.

**Methods:**

We searched seven databases on 1 September 2019 using search terms for ethnicity, dementia, and incidence or prevalence. We included population-based studies comparing incidence or prevalence of dementia after accounting for age of at least two ethnic groups in adults aged 18 or more. Meta-analysis was conducted for eligible ethnic comparisons.

**Results:**

We included 12 cohort studies and seven cross-sectional studies. Thirteen were from the US, and two studies each from the UK, Singapore, and Xinjiang Uyghur Autonomous Region in China. The pooled risk ratio for dementia incidence obtained from four studies comparing Black and White ethnic groups was 1.33 (95% CI 1.07–1.65; I-squared = 58.0%). The pooled risk ratio for dementia incidence comparing the Asian and White ethnic groups was 0.86 (95% CI 0.728–1.01; I-squared = 43.9%). There was no difference in the incidence of dementia for Latino ethnic group compared to the White ethnic group.

**Conclusion:**

Evidence to date suggest there are ethnic differences in risk of dementia. Better understanding of the drivers of these differences may inform efforts to prevent or treat dementia.

## INTRODUCTION

Globally around 50 million people have dementia, and this number is projected to reach 75 million by 2030. The burden of dementia is growing rapidly in low- and middle-income countries (LMICs). More than $800 billion is spent worldwide on caring for people with dementia every year and this is set to reach $2 trillion by 2030 [1]. A UK study estimated that the health and social care costs on dementia are higher than that on cancer, stroke, and heart disease combined [2].

Some dementia risk factors such as hypertension and diabetes are more common in Black, Asian, and other ethnic minorities [3–5]. There are also marked ethnic inequalities in care seeking, likelihood of diagnosis, and uptake of treatments for dementia [6–8]. While ethnic differences in incidence and prevalence have been most closely studied in the USA, there are still substantial knowledge gaps about dementia incidence and prevalence among diverse ethnic groups globally [9].

A large recent systematic review reported higher dementia incidence in African Americans and Caribbean Hispanic Americans compared to the White ethnic group [10]. However, this review was limited to the US, and included studies where the results were not age-specific or age-adjusted. A cohort study using routine healthcare data from the UK looked at ethnic differences in dementia incidence and reported higher incidence dementia in Black men and women compared to White men and women [8]. A cohort study using routine healthcare data from the UK looked at ethnic differences in incidence of diagnosed dementia after stroke and reported higher incidence in the Black ethnic group compared to the White ethnic group [11]. However, it is unclear whether these differences are also observed globally, and the relative contribution of biological and socio-cultural factors is unclear. These could also be due to differences in recording as only about two-thirds of dementia cases are identified in routine data [12] and cohort studies might lead to different results.

We conducted this systematic review to examine previously published population-based studies that compared incidence and prevalence rates across ethnic groups globally, and meta-analyzed where possible. Specific objectives were to examine published peer-reviewed observational studies conducted on adults over 18 years of age comparing age-specific or age-adjusted incidence or prevalence rates of 1) All-cause Dementia, and 2) Dementia sub-types, e.g., Alzheimer’s disease dementia and vascular dementia, between at least two ethnic groups. Identifying such differences could help inform research into preventative interventions and service provision for these groups.

## METHODS

### Protocol and registration

We conducted this systematic review according to the Preferred Reporting Items for Systematic Review and Meta-analysis (PRISMA) statement [13]. Our protocol is available on the PROSPERO [14] database under the registration number CRD42019133385.

### Eligibility criteria

We did not restrict studies by geographical location or language.

#### Inclusion

Participants: Studies including adults who are at least 18 years old.

Exposure: Any ethnicity, e.g., Black, White, South Asian, etc.

Comparator: Studies comparing at least two different ethnic groups in the same population.

Outcomes: The main outcome was age-specific or age-adjusted prevalence or incidence of clinically diagnosed or self-reported dementia of any type. Additional outcomes included age-specific or age-adjusted prevalence or incidence of clinically diagnosed or self-reported dementia sub-types, such as Alzheimer’s disease dementia and vascular dementia. Effect measures such as odds ratios, risk ratios, and hazard ratios were presented.

Studies: We only included published peer-revie-wed articles from population-based studies, e.g., co-hort (traditional and routine data based), case-control, cross-sectional.

#### Exclusion

Studies with delirium, acute encephalopathy, or mild cognitive impairment as outcome.

Studies with less than 100 people to exclude potentially underpowered studies.

### Information sources

We conducted a systematic review of literature using the databases MEDLINE, EMBASE, Psyc-INFO, CINAHL, Scopus, Global Health, and Web of Science. We searched these databases from inception to 1 September 2019.

### Search

To identify studies on ethnic differences in dementia incidence and prevalence, we used a combination of subject heading and keyword searches for incidence and prevalence, dementia and its sub-types, and ethnicity, and combined these using the operator AND. Detailed searches for each database are in the Supplementary Material. We also hand searched reference lists of included systematic reviews for additional articles.

### Study selection

Search results from all databases were exported into EndNote X8, where duplicates and non-peer reviewed items were removed (e.g., book references and conference abstracts). Title and abstracts of remaining references were independently screened by two reviewers (SS and SC) using Covidence software [15, 16], to identify potentially relevant articles.

### Full text review

Both SS and SC reviewed potentially relevant full-text articles against the inclusion and exclusion criteria. We also screened the reference lists of review papers related to our study for additional studies that met our eligibility criteria. Reasons for excluding papers at this stage were agreed upon when there were discrepancies and documented. A third reviewer (CWG) was available to adjudicate in case of any discrepancies that could not be resolved by SS and SC.

### Data collection process

We developed a data extraction form in Windows Excel. SS and SC pilot tested the template using a 10% random sample of the studies and finalized it. We extracted the following information:

• Publication: first author and publication year
• Study characteristics and population: setting, design, aims and objectives, study period, recruitment and sampling methods, language, study population at recruitment
• Ethnicity exposure: definition and ascertainment
• Comparators: definition and ascertainment
• Outcomes: types, definition and ascertainment, whether incident or prevalent
• Results: population size, follow-up time, study population characteristics, subject with outcome, statistical analysis methods used, main reported crude and adjusted results, confounders measured and adjusted for.

SS extracted the data for all the studies and SC extracted data for a 10% random sample of the studies. One of the authors (LL) who was fluent in Mandarin extracted data from two studies in Mandarin.

### Risk of bias in individual studies

SS assessed the risk of bias of all included studies and SC assessed this for 10% of studies. We incorporated domains relevant to observational studies in risk of bias assessment tools like the Newcastle-Ottawa Scale and ROBINS-I to develop our risk of bias assessment template. Our template included domains on confounding, selection bias, study power, misclassification of exposure, outcome, and covariates, handling of missing data, and generalizability. We also included a domain for reverse causation for completeness to enable comparison of risk of bias assessments conducted for studies of other dementia risk factors.

### Summary measures

The principal summary measures included were risk ratios, rate ratios, hazard ratios, odds ratios, and incidence and prevalence rates.

### Synthesis of results

We grouped studies by pairs of ethnic groups being compared and the type of outcome reported in more than one of the included studies. For instance, we grouped studies reporting results from incident dementia and comparing White and Black ethnic groups. Similarly, other comparison groups were White and Asian and White and Latino for studies with incident dementia as the outcome, and Chinese and Malay and Chinese and Indian for studies with prevalent dementia as the outcome. We made comparisons within each group when we had summary measures which were directly comparable using Forests plots to visualize results. We generated pooled estimates using meta-analysis with random-effects for comparisons of White with Black and Asian ethnic groups for incident outcomes, and Chinese with Malay and Indian ethnic groups for prevalent outcomes.

We grouped ethnicities reported as White, Non-Latino White, and European American as White; Black, African American, and African-Caribbean as Black; Asian and Asian-American as Asian; and Latino, Hispanic, Caribbean Hispanic, and Cuban Americans as Latino. Other ethnic groups included in this review were American Indian or Alaskan Native, Pacific Islander, Chinese, Malay, Indian, Han, Kazakh, Uyghur, Japanese, Filipino, South Asian, Other/Unknown Asian, and Mixed.

To facilitate comparison with other studies, we changed the reference ethnic group from Asian to White to calculate Hazard ratio (HR) effect estimates for the ethnic groups included using the results reported in Mayeda et al. [17]. However, since the covariances between the original parameters were not available, we could only calculate the 95% confidence intervals for the Asian ethnic group. We also calculated the rate ratio using the incidence rates reported in Mayeda et al. [18] for the All Asians and White ethnic group categories. We also calculated the rate ratio comparing White and Black ethnic groups from the incidence rates reported by Fitzpatrick et al. [19].

### Risk of bias across studies

We were unable to assess publication bias as the number of studies in each comparison (e.g., White and Black, and White and Asian comparisons) was insufficient to conduct reliable tests for funnel plot symmetry [20].

## RESULTS

**Fig. 1:**
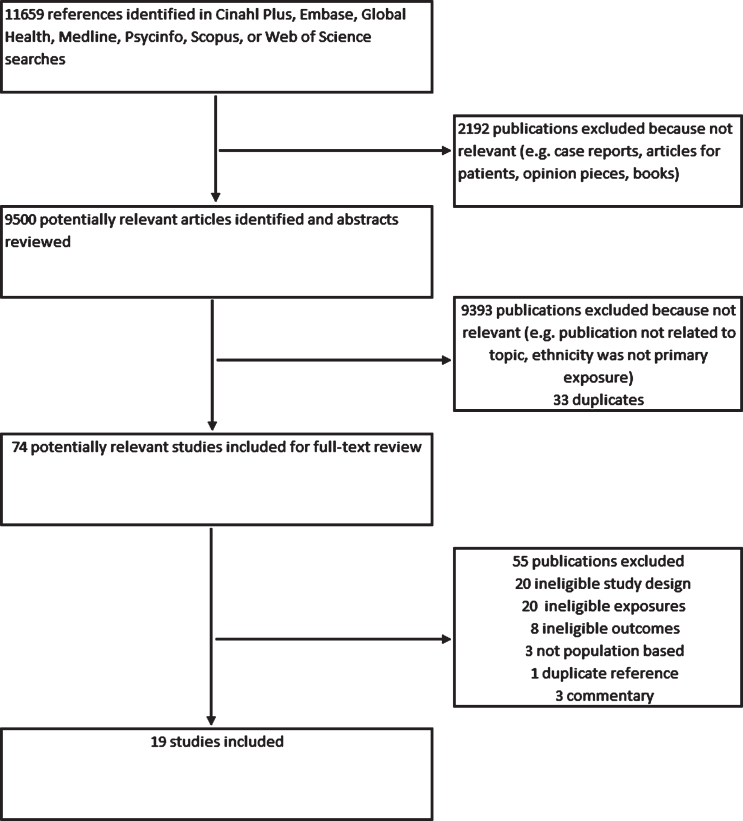
Flowchart of search stages.

We identified 11,659 articles in the initial search after removing duplicates. After removing non-peer reviewed items, we screened title and abstracts for 9,500 articles. We reviewed full texts for 74 of these and included 19 studies in our review (Fig. 1).

### Study characteristics and findings

Of the 19 included studies, 12 were cohort studies and seven were cross-sectional studies (Table 1). Thirteen studies were from the US, of which 10 were primary research studies in the community and three were based on electronic health records. We had two studies from the UK out of which one was a primary research study and one was based on electronic health records. We also had two primary research studies each from Singapore and the Xinjiang Uyghur Autonomous Region in China. We had 11 cohort studies from the US and one from the UK. All studies other than the two Chinese studies were in English. Of the 19 studies, nine studies assessed incident dementia as the outcome while 10 studies looked at prevalent dementia. Fourteen studies had any dementia as their main outcome while five had dementia sub-types, Alzheimer’s disease dementia and vascular dementia, as their main outcome (Table 2).

**Table 1:** Study Characteristics and Population

First author, publication year	Setting	Design	Study period	Recruitment and sampling methods	Language	Study population at recruitment	Population size (N), follow-up time (years)	Study population characteristics
Gilsanz 2019 [21]	US, EHR	Cohort	2010–2015	EHR	English	90 years or older, included in KPNC database, mean age at baseline- 93.1 (SD 2.6) years.	White 1,702 Black 373 Latino 105 Asian 168	2010
								White mean age (SD) 93.1 (2.6), % male 33.8
								Black mean age (SD) 92.9 (2.5), % male 35.2
								Latino mean age (SD) 92.8 (2.8), % male 31.4
								Asian mean age (SD) 92.8(2.5), % male 44.1
Katz 2012 [22]	US, community	Cohort	1993-unclear	Random	English	70 years or older, Bronx community residents, English speaking; Exclusion criteria: inability to complete assessments visual due to auditory impairments, active psychiatric symptomatology, or non-ambulatory status	1,168 White 817 Black unclear average follow-up of 3.9 years	1944 total, mean age of cohort at baseline 78.8 years, 39.3% male
Mayeda 2016 [17]	US, EHR	Cohort	2000–2013	EHR	English	65 years or older, included in KPNC database, no dementia diagnosis at study onset	274,283 participants	At baseline, mean age was 73.4
							AA 18,778	years and 45.4% was male
							AIAM 4,543	mean age (SD), % male
							Latino 21,000	AA 72.7 (6.5), 45.1%
							Pacific Islander 440	AIAN 73.5 (6.3, 45.7%
							White 206,490	Latino 71.9 (5.9), 47.6%
							Asian-American 23,032	Pacific Islander 71.5 (7.0), 50.2%
							mean follow-up of 8.6 years (SD 4.9)	White 73.9 (6.7), 45.1% Asian-American 71.7(5.9), 46.9%
							29.0% died, 17.0% lost to follow-up
Pham 2018 [8]	UK, EHR	Cohort	2007–2015	EHR	English	50 years or older, included in THIN database	2,511,681 total	Number
							median age	White–1,112,840
							(IQR)–59.5 (51.5–70.5)	Asian–31,757
							% male–48%	Black–18,214
							median follow-up (IQR) 5.5 years (2.6–8.6)	Mixed/Other–15,300
							Missing–1,333,570
Weuve 2018 [23]	US, community	Cohort	1993–2012	Stratified random	English	65 and older, from South Chicago	2,909 clinical evaluations
Fitzpatrick 2004 [19]	US, community	Cohort	1989–1999	Random	English	65 and older, from four U.S. communities	3,602 total	492 (14.6%) of the cohort were AA, and 40.9% were men
							White 2,865
							AA 492
							mean follow-up of 5.4 years
Chen 2018 [26]	US, community	Cohort	2000–2012	Stratified random	English	65 years and older, living in community or in nursing homes	18,606 total	Overall White 84.9%, Black
							2000:	8.6%, Hispanic 6.5%
							White 8,474	2000:
							Black 1,337	White 41.6% male
							Hispanic 738:	Black 38.0% male
							2012:	Hispanic 42.5% male
							White 8,067	2012
							Black 1,478	White 44.0% male
							Hispanic 975	Black 39.1% male
							average follow-up of four waves	Hispanic 41.8% male
Adelman, 2011 [28]	England, community	Cross-sectional prevalence study	Unclear	Simple random	English	60 years and over, from Haringey, living in the community or in nursing homes	White UK-born 218 AC 2018 follow-up time N/A	AC: mean age 71.8, 39.4% male White: mean age 73.7, 41.3% male
Gurland 1999 [25]	US, community or nursing home records	Cohort	1989–1991	Random	English	65 years or older, from 13 adjacent census tracts in North Manhattan	Baseline	Baseline
							Latino 1,001	Latino, 30.6% male
							AA 729	AA, 28.5% male
							NLW 432	NLW, 33.3% male
							1st Follow-up	1st follow-up
							Latino 693	Latino, 29.7% male
							AA 454	AA, 29.3% male
							NLW 267	NLW, 35.2% male
							Average follow-up 18 months
Ng 2010 [32]	Singapore, community	Cross-sectional prevalence study	2003–2004	Random	English	60 years or older, Singaporean citizens or permanent residents	1,092 total mean age 69.4 (SD 7.1)	Ethnicity-number, mean age (SD), % male
								Chinese–501, 69.8 (7.6), 42.7%
								Malay–354, 69.0 (6.7), 44.6%
								India–237, 69.0 (6.5), 51.9%
Sahadevan 2008 [33]	Singapore, community	Cross-sectional prevalence study	2001–2003	Stratified random	English	50 and older, Singaporean citizens or permanent residents	14,817 total 45.3% male	Chinese 8849, 44.8% male Malay 3053, 45.3% male Indian 2915, 46.7% male
Yaffe 2013 [24]	US, community	Cohort	1997–2011	Stratified random	English	70–79 years, living in community	2457 participants	% male
							Black 1019	Black 41.5%
							White 1438	White 58.5%
								baseline mean age 73.6 years
Mayeda 2017 [18]	US, EHR	Cohort	2000–2013	EHR	English	65 years or older, included in KPNC database, no dementia diagnosis at study onset	Whites	Baseline mean age, % male
							206,490	White 73.9, 45.1%
							Chinese 8,384	Chinese 71.9, 51.7%
							Japanese 4,478	Japanese 72.4, 36.1%
							Filipino 6,210	Filipino 71.4, 46.0%
							South Asian 197	South Asian 69.8, 73.1%
							Other/Unknown Asian 3,763	Other/Unknown Asian 71.0, 49.3%
								mean follow-up 9.6 years
Moon 2019 [27]	US, community	Cross-sectional prevalence study	2011	Stratified random	English	Medicare beneficiaries 65 and older	7,609 participants	% male
							NHW 5,185	Total 43.4%
							NHB 1,660	NHW 43.6%
							Hispanic 454	NHB 39.7%
							Others 149	Hispanic 44.2%
								Others 42.8%
Demirovic 2003 [31]	US, community	Cross-sectional prevalence study	1993–1997	Neighborhoods selection unclear, other stages random	English	65 years or older, from three homogenous ethnic neighborhoods in Miami Dade County	2,759 total WNH 942 AA 827 CA 990 follow-up time N/A	NHW –343 male (mean age 77.9), 599 female (mean age 78.8)
								AA –289 male (mean age 73.3),
								538 female (mean age 73.7)
								CA –374 male (mean age 75.1), 616 female (mean age 75.7)
Rajan 2019 [30]	US, community	Cohort	1994–2012	Stratified random	English	65 and older, from South Chicago	2,794 total mean age (SD) 76.2 (0.20) 35% male	Number, mean age (SD), % male AA–1,561, 75.6 (0.25), 34% EA–1,233, 77.0 (0.32), 37%
Meng 2014 [44]	Xinjiang Uyghur Auto-nomous Region, China	Cross-sectional prevalence study	2010–2012	Stratified cluster random	Simplified Chinese	55 years or older, living in Xinjiang in 2010 census	3,663 total	Kazak people: *N* = 2532,
								M: 1,221 (48.22%), mean age: 69.46 (SD 7.85)
								F: 1,311 (51.78%), mean age: 68.67 (SD 7.14)
								Han people: *N* = 1078
								M: 501(46.47%), mean age: 70.16 (7.13)
								F: 589 (53.53%), mean age: 68.72 (7.79)
Zhou 2008 [45]	Xinjiang Uyghur Auto-nomous Region, China	Cross-sectional prevalence study	2004–2007	Stratified cluster random	Simplified Chinese	50 years or older	8,284 total	Uyghur ethnic people: *n* = 4,688,
								M: 2,324, mean age: 65 (10)
								F: 2,364, mean age: 62.6 (9)
								Han ethnic people: *n* = 3,596
								M: 1,592, mean age: 64.1 (8)
								F: 2,004, mean age: 61.7 (7.8)
Tang 2001 [29]	US, community	Cohort	1992–1999	Random	English	65 years or older, from three contiguous census tracts in northern Manhattan	1,799 total follow-up duration (SD) White 4.3 (1.5) Black 4.3 (1.5) Caribbean Hispanic 4.4 (1.4)	Sample proportion, Male % White 23.4%, 35% Black 43.1%, 29% Caribbean Hispanic 42.5%, 32%

HR, hazard ratio; RR, risk (or rate) ratio; IRR, incidence rate ratio; CI, confidence interval; AD, Alzheimer’s disease dementia; VaD, vascular dementia; OR, odds ratio if specified; EHR, electronic health records; AA, African-American; AIAN, American Indian and Alaskan Native; AC, African-Caribbean; CA, Cuban American; NHB, Non-Hispanic Black; NHW, Non-Hispanic White; NLW, Non-Latino White.

**Table 2:** Outcomes and Results

First author, publication year	Outcome	Definition and ascertainment	Incidence/Prevalent	Statistical analysis method used	Main reported adjusted results	Adjusted for
Gilsanz 2019 [21]	Dementia	From electronic health records	Incident	Cox proportional hazards models	Hazard Ratios for Dementia (95% CI)	Age, sex, BMI, educational attainment, hypertension, hypercholesterolemia, depression, diabetes, stroke, ischemic heart disease, and heart failure
					White 1
					Black 1.28 (1.06, 1.56)
					Latino 1.12 (0.79, 1.59)
					Asian 1.00 (0.75, 1.34)
Katz 2012 [22]	Dementia	Clinical examinations and neuro-cognitive tests	Incident	Cox proportional hazards models	Hazard Ratios for	Age, sex, education
					Dementia (95% CI)
					Whites 1
					Blacks 1.31 (0.88, 1.94)
Mayeda 2016 [17]	Dementia	From electronic health records	Incident	Cox proportional hazards models	HR (95% CI)	Age, sex, health care utilization, depression, diabetes, hypertension, stroke, and cardiovascular disease.
					AA 1.65 (1.58, 1.72)
					AIAN 1.32 (1.24, 1.41)
					Latino 1.24 (1.19, 1.29)
					Pacific Islander 1.23 (0.95, 1.58)
					White 1.22 (1.18, 1.26)
					Asian-American 1
Pham 2018 [8]	Dementia	From electronic health records	Incident	Poisson regression models	IRR (95% CI)	Age, calendar year, Townsend deprivation score, prescribing index, diabetes, stratified by sex
					Men
					White 1
					Asian 0.88 (0.76, 1.01)
					Black 1.28 (1.08, 1.50)
					Mixed/Other 0.86 (0.69, 1.08)
					Women
					White 1
					Asian 0.82 (0.72, 0.95)
					Black 1.25 (1.07, 1.46)
					Mixed/Other 0.97 (0.08, 1.18)
Weuve 2018 [23]	Dementia, AD	Clinical examinations and neuro-cognitive tests	Incident	Logistic regression models, weighted for the stratified random sampling design, variance parameters computed by jack-knife repeated replication	Risk ratio (95% CI)	Age, sex
					All-cause dementia
					Black 1.99 (1.27, 2.71)
					White 1
					AD
					Black 2.04 (1.26, 2.82)
					White 1
Fitzpatrick 2004 [19]	Dementia	Clinical examinations and neuro-cognitive tests	Incident	Cox proportional hazards models	Age-adjusted (at age 80)	Age
					Incidence of dementia (per
					1,000 Person-years)
					White
					Male 35.3
					Female 34.7
					Total 32.7 (*p* = 0.003)
					Black
					Male 53.0
					Female 58.8
					Total 56.4 (*p* = 0.003)
Chen 2018 [26]	Dementia	Clinical examinations and neuro-cognitive tests	Prevalent	Mixed-effects logistic regression with both intercept and time trend to vary by individuals, using random-effects unstructured covariance to control for repeated observations	2000–2012	Biennial trend, age, gender, stroke, hypertension, diabetes, heart disease, BMI, vigorous activity, education, wealth
					OR (95% CI)
					White 1
					Black 2.18 [1.91, 2.49]
					Hispanic 1.47 (1.25, 1.73)
Adelman, 2011 [28]	Dementia	Clinical examinations and neuro-cognitive tests	Prevalent	Logistic regression	OR (95% CI) AC 3.07 (1.28, 7.3) White 1	Age, socio-economic status
Gurland 1999 [25]	Dementia	Clinical examinations and neuro-cognitive tests, weighting, algorithm	Prevalent		Prevalence of dementia in the three age strata based on criterion diagnosis	Age-specific prevalence rates presented by groups
					Latino
					65–74 7.5%
					75–84 27.9%
					85 + 62.9%
					AA
					65–74 9.1%
					75–84 19.9%
					85 + 58.6%
					NLW
					65–74 2.9%
					75–84 10.9%
					85 + 30.2%
Ng 2010 [32]	Dementia	Clinical examinations and neuro-cognitive tests	Prevalent	Logistic regression	OR (95% CI)	Age, gender, education
					Chinese 1
					Malay 3.11 (1.68, 5.77)
					Indian 4.30 (2.13, 8.71)
Sahadevan 2008 [33]	AD, VaD	Clinical examinations and neuro-cognitive tests	Prevalent	Logistic regression	OR (95% CI)	Age, sex, education
					Any dementia
					Chinese 1
					Malay 1.92 (1.35, 2.72)
					Indian 2.32 (1.57, 3.42)
					AD
					Chinese 1
					Malay 2.18 (1.38, 3.44)
					Indian 2.28 (1.34, 3.88)
					VaD
					Chinese 1
					Malay 1.45 (0.83, 2.55)
					Indian 2.19 (1.24, 3.85)
Yaffe 2013 [24]	Dementia	Clinical examinations and neuro-cognitive tests	Incident	Cox proportional hazards models	HR (95% CI) White 1 Black 1.09 (0.87, 1.37)	Age, sex, apolipoprotein E *ɛ*4, comorbidities, lifestyle, and socioeconomic measures
Mayeda 2017 [18]	Dementia	From electronic health records	Incident	Cox proportional hazards models	HR (95% CI)	Age, sex, healthcare utilization (≥1 healthcare visit per year), comorbidities (depression, diabetes, hypertension, stroke, and CVD)
					Chinese 1
					Japanese 1.08 (0.99, 1.18)
					Filipino 1.20 (1.11, 1.31)
					SA 0.81 (0.53, 1.25)
					Other/Unknown Asian 1.22 (1.10, 1.34)
					Age-standardized incidence rate (95% CI)
					White 19.35 (19.16, 19.54)
					Chinese 13.67 (12.92, 14.42)
					Japanese 14.80 (13.74, 15.86)
					Filipino 17.26 (16.15, 18.38)
					SA 12.09 (6.10, 18.07)
					Other/Unknown Asian 16.73 (15.25, 18.21)
Moon 2019 [27]	Probable dementia	From electronic health records	Prevalent	Log-binominal analyses	Relative Risk (95% CI)	Age, sex, education, number of people in household, number of cardiovascular conditions, immigrant status
					NHW 1
					NHB 1.465 (1.277, 1.681)
					Hispanic 1.154 (0.862, 1.544)
					Others 1.455 (1.055, 2.007)
Demirovic 2003 [31]	AD	Clinical examinations and neuro-cognitive tests	Prevalent	Multiple logistic regression	OR (95% CI) AD:	Age, sex, education, cigarette smoking, alcohol use, marital status, history of hypertension, history of head trauma, family history of AD
					NHW 1
					AA 7.38 (3.23, 16.63)
					CA 3.17 (1.39, 7.2)
Rajan 2019 [30]	AD	Clinical examinations and neuro-cognitive tests	Prevalent		Prevalence 2010–2012	Age
					AD:
					AA 30.0 (26.6, 33.5)
					EA 14.9 (12.6, 17.1)
Meng 2014 [44]	AD, VaD,	Clinical examinations and neuro-cognitive tests translated into Kazak or Mandarin	Prevalent	Standardized rate and Chi-square	Age-adjusted standardized prevalence:	Age
					AD:
					Kazak people: 5.64%
					Han: 4.73%
					VaD:
					Kazak people: 2.43%
					Han: 1.99%
Zhou 2008 [45]	AD, VaD,	Clinical examinations and neuro-cognitive tests	Prevalent	Standardized rate and Chi-square	Age-adjusted standardized prevalence:	Age
					AD:
					Uyghur people: 2.68%
					Han: 4.31%
					VaD:
					Uyghur people: 1.00%
					Han: 0.96%
Tang 2001 [29]	AD	Clinical examinations and neuro-cognitive tests	Incident	Cox proportional hazards models	HR (95% CI) AD:	Age, hypertension, heart disease, stroke, diabetes, and years of education
					White 1
					Black 2.4 (1.5, 4.0)
					Caribbean Hispanic 2.0 (1.2, 3.4)

HR, hazard ratio; RR, risk (or rate) ratio; IRR, incidence rate ratio; CI, confidence interval; AD, Alzheimer’s disease dementia; VaD, vascular dementia; OR, odds ratio if specified; EHR, electronic health records; AA, African-American; AIAN, American Indian and Alaskan Native; AC, African-Caribbean; CA, Cuban American; NHB, Non-Hispanic Black; NHW, Non-Hispanic White; NLW, Non-Latino White.

Study populations ranged from 1,092 to 2,511,681 individuals. Dementia was diagnosed using clinical examinations and neuro-cognitive tests in all studies although it was ascertained using electronic health records in four studies. Mean baseline age ranges were from 62 to 93 years in nine studies where this information was available.

### Comparison of White and Black ethnic groups

Thirteen studies featured comparisons of White and Black ethnic groups (twelve from the USA and one from the UK). Of these, 7 compared all-cause dementia incidence, three compared all-cause dementia prevalence, one compared Alzheimer’s disease dementia incidence, and two compared Alzhei-mer’s disease dementia prevalence.

**Fig. 2:**
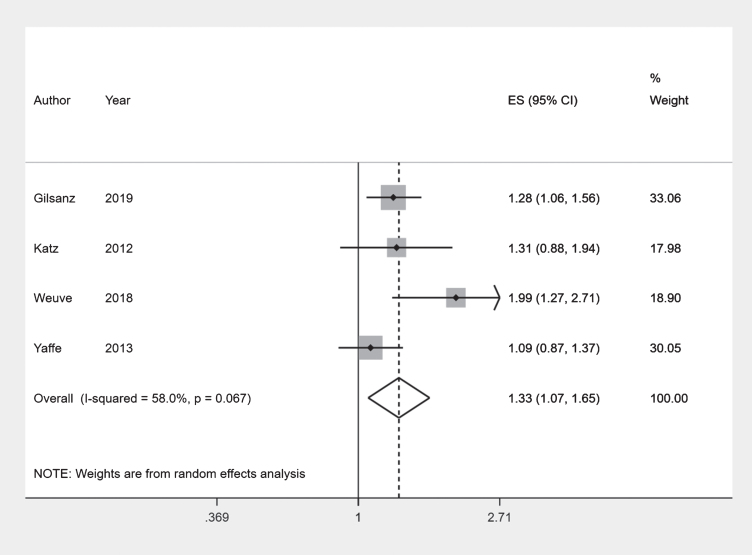
Forest plot: White and Black ethnic groups.

Overall, the Black ethnic group had higher incidence rates than the White ethnic group. Combining results of four comparable incident outcome studies [21–24] using a meta-analysis we obtained a pooled risk ratio of 1.33 (95% CI 1.07–1.65) with an I-squared value of 58.0% (Fig. 2). This was similar to results reported by Pham et al. [8] by sex [Men: Incidence Rate Ratio (IRR) 1.28 (95% CI 1.08–1.50); Women: IRR 1.25 (95% CI 1.07–1.46)], and the rate ratios calculated for Mayeda et al. [17] (1.35) and Fitzpatrick et al. [19] (1.72) which could not be included in the meta-analysis due to the differences in their reported summary measures (Table 2).

Results from four included studies examining prevalent dementia reported the risk or odds of prevalent dementia between 1.47 to 3.01 times higher in the Black ethnic groups compared to the White ethnic group. The prevalence in the 65–74 years age group was over 3 times higher in Gurland et al. [25]. Comparing Black and White ethnic groups Chen and Zissimopoulos [26] reported an odds ratio (OR) of 2.18 (95% CI 1.91–2.49) while Moon et al. [27] reported a relative risk (RR) of 1.47 (95% CI 1.28–1.68). Adelman et al. [28] reported an odds ratio (OR) of 3.01 (95% CI 1.28–7.3) comparing African-Caribbean group to the White ethnic group (Table 2).

Similarly, the Black ethnic group had more than twice the incidence rate for Alzheimer’s disease dementia compared to the White ethnic group according to Tang et al. [29] [HR 2.4 (95% CI 1.5–4.0)]. The Black ethnic group had two times the prevalence rate for Alzheimer’s disease dementia compared to the White ethnic group according to Rajan et al. [30]. Demirovic et al. [31] reported that the Black ethnic group had 7.38 (95% CI 3.23–16.63) times the odds of having Alzheimer’s disease dementia compared to the White ethnic group (Table 2).

#### Risk of bias assessment: White and Black ethnic groups

The studies looking at incident dementia had low risk of bias for most of the assessed criteria. However, Gilsanz et al. [21] had a high risk of bias for two criteria, misclassification of variables and power. The studies looking at prevalence of dementia had low to moderate risk of bias for most of the categories, although Moon et al. [27] had high risk of bias for two criteria. Studies looking at Alzheimer’s disease dementia as the outcome had moderate to high risk for most criteria (Table 3).

**Table 3:** Risk of bias assessment summary

First author, publication year	Confounding	Selection of participants	Misclassification of variables	Bias due to missing data	Reverse Causation	Power
*Any dementia*
Gilsanz 2019 [21]	•	•	◊	•	•	◊
Katz 2012 [22]	•	•	■	∘	•	◊
Mayeda 2016 [17]	•	•	◊	•	•	•
Pham 2018 [8]	•	•	◊	•	•	•
Weuve 2018 [23]	•	•	•	∘	•	◊
Fitzpatrick 2004 [19]	■	•	■	∘	•	◊
Chen 2018 [26]	•	•	■	•	•	•
Adelman, 2011 [28]	•	•	•	•	•	•
Gurland 1999 [25]	■	■	◊	•	•	◊
Ng 2010 [32]	•	■	■	•	•	◊
Sahadevan 2008 [33]	•	■	■	•	•	◊
Yaffe 2013 [24]	•	•	■	•	•	◊
Mayeda 2017 [18]	•	•	◊	•	•	•
Moon 2019 [27]	•	•	◊	•	•	◊
*Dementia sub-types*
Demirovic 2003 [31]	•	◊	◊	•	•	◊
Rajan 2019 [30]	■	•	•	■	•	◊
Meng, 2014 [44]	■	•	◊	•	•	◊
Zhou, 2008 [45]	■	•	∘	•	•	◊
Tang 2001 [29]	•	•	■	■	•	◊

◊High risk. ■Moderate Risk. •Low Risk. ∘Unclear. Note: More detailed risk of bias assessment in Supplementary Table 1.

### Comparison of White and Asian ethnic groups

Four studies featured comparisons of all-cause dementia incidence between White and Asian ethnic groups (three from the US and one from the UK).

**Fig. 3:**
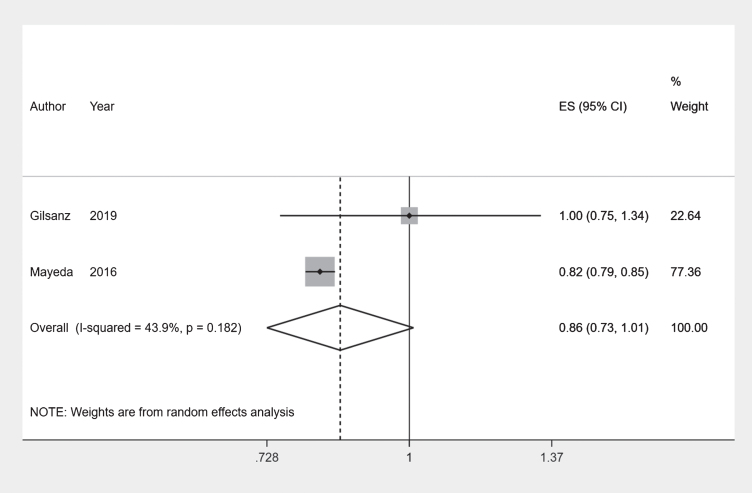
Forest plot: White and Asian ethnic groups.

Overall, the incidence rates of the Asian ethnic group did not appear to be different from that of the White ethnic group. Combining results of two comparable incident outcome studies [17, 21] using a meta-analysis we obtained a pooled risk ratio of 0.86 (95% CI 0.728–1.01) with an I-squared value of 43.9% (Fig. 3). Although the point estimate suggests a protective effect, the confidence interval just crosses the null value. This was similar to results reported by Pham et al. [8] by sex [Men: IRR 0.88 (95% CI 0.76–1.01); Women: IRR 0.82 (95% CI 0.72–0.95)]. Mayeda et al. [18] reported lower age-standardized incidence rates for all Asian ethnic sub-groups compared to the White ethnic group (Rate Ratio = 0.79) (Table 2).

#### Risk of bias assessment: White and Asian ethnic groups

The studies looking at incident dementia had low risk of bias for most of the assessed criteria, although Gilsanz et al. [21] had a high risk of bias for two criteria (Table 3).

### Comparison of White and Latino ethnic groups

Five studies featured comparisons of White and Latino ethnic groups (all five from the US). Of these, two compared all-cause dementia incidence, three compared all-cause dementia prevalence, and one study compared Alzheimer’s disease dementia incidence and prevalence each.

The incidence rates of dementia for the Latino ethnic group did not appear to be different from that of the White ethnic group. Gilsanz et al. [21] reported a hazard ratio (HR) of 1.12 (95% CI 0.79–1.59) comparing the Latino and White ethnic groups, while the calculated HR from Mayeda et al. [17] was 1.02 between the same groups (Table 2).

Gurland et al. [25] reported over two times the prevalence rates for any dementia comparing the Latino ethnic group to the White ethnic group for all age-groups. Chen and Zissimopoulos [26] reported an odds ratio (OR) of 1.47 (95% CI 1.25–1.73) while Moon et al. [27] reported a relative risk (RR) of 1.15 (95% CI 0.86–1.54) comparing Latino and White ethnic groups (Table 2).

Tang et al. [29] reported a hazard ratio of 2.0 (95% CI 1.2–3.4) comparing Caribbean Hispanic and White ethnic groups for Alzheimer’s disease dementia. Demirovic et al. [31] reported that the Cuban Americans had 3.17 (95% CI 1.39–7.20) times the odds of having Alzheimer’s disease dementia compared to the White ethnic group (Table 2).

#### Risk of bias assessment: White and Latino ethnic groups

Of the two studies looking at incident dementia Gilsanz et al. [21] had a high risk of bias for two criteria while Mayeda et al. [17] had low risk for all criteria except one. Most studies looking at prevalence of dementia had high risk of bias for at least two categories, although Chen and Zissimopoulos [26] had low risk of bias for all criteria except one. Of the studies looking at Alzheimer’s disease dementia as the outcome Demirovic et al. [31] had high risk of bias for more than two criteria while Tang et al. [29] had low to moderate risk for most criteria (Table 3).

### Comparison of Chinese and Malay ethnic groups

**Fig. 4:**
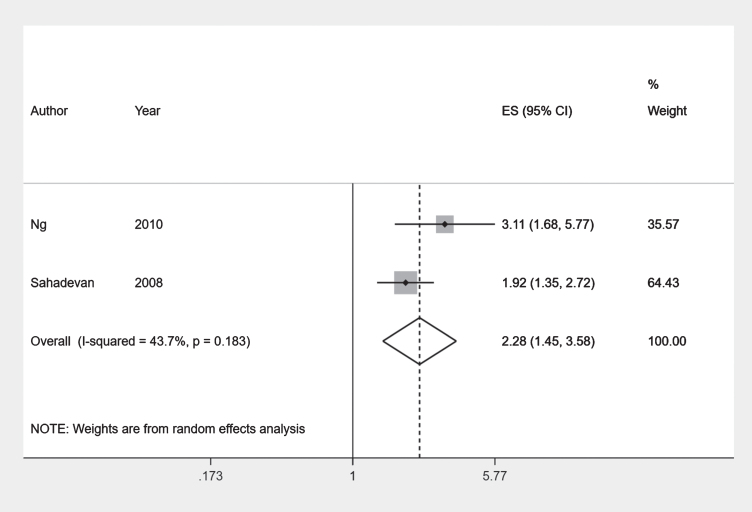
Forest plot: Chinese and Malay ethnic groups.

Overall, the Malay ethnic had higher odds of having the any dementia outcome compared to the Chinese ethnic group. Combining results of two comparable prevalent outcome studies [32, 33] using a meta-analysis we obtained a pooled odds ratio of 2.28 (95% CI 1.45–3.58) with an I-squared value of 43.7% (Fig. 4).

#### Risk of bias assessment: Chinese and Malay ethnic groups

The two cross-sectional prevalence studies [32, 33] had low to moderate risk of bias for most criteria (Table 3).

### Comparison of Chinese and Indian ethnic groups

**Fig. 5:**
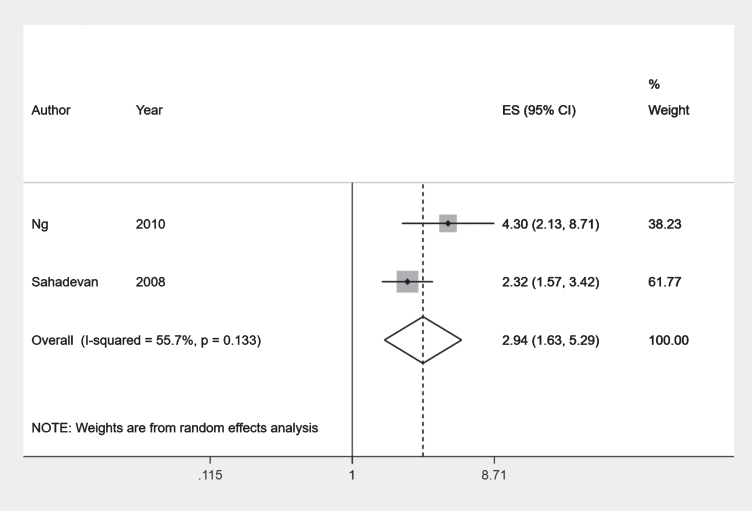
Forest plot: Chinese and Indian ethnic groups.

Mayeda et al. [18] reported a hazard ratio of 0.81 (95% CI 0.53–1.25) for incidence of dementia in South Asians compared to Chinese ethnic group. Overall, the Indian ethnic group had higher odds of having the any dementia outcome compared to the Chinese ethnic group. Combining results of two comparable prevalent outcome studies [32, 33] using a meta-analysis we obtained a pooled odds ratio of 2.94 (95% CI 1.63–5.29) with an I-squared value of 55.7% (Fig. 5).

#### Risk of bias assessment: Chinese and Indian ethnic groups

The two cross-sectional prevalence studies [32, 33] had low to moderate risk of bias for most criteria (Table 3).

### Other comparisons

We included results from individual studies comparing other pairs of ethnic groups not in the above categories in Table 2.

## DISCUSSION

Our systematic review included 19 studies from four settings: the UK, the US, Singapore, and the Xinjiang Uyghur Autonomous Region in China. The evidence comparing the incidence of dementia in the Black ethnic group compared to the White ethnic was from longitudinal studies and of acceptable quality. Of the four studies included in the meta-analysis, Gilsanz et al. [21] was based on electronic health records with similar results to the primary research studies in the community, while two of the three studies in the narrative synthesis were based on electronic health records. Overall, these suggested that the Black ethnic group have higher incidence of dementia compared to the White ethnic group. There was also some evidence of higher prevalence of dementia, and incidence and prevalence of Alzheimer’s disease dementia in the Black ethnic group compared to the White ethnic group. The evidence from studies with similarly grouped ethnicities looking at incidence of dementia in the Asian and Latino ethnic groups compared to the White ethnic group was from longitudinal studies based on electronic health records and of acceptable quality. While there was a suggestion of lower incidence in Asians compared to the White ethnic group, the confidence intervals just cross the null value. There did not appear to be a difference in dementia incidence comparing the Latino ethnic group with the White ethnic group in the two longitudinal studies based on electronic health records. The evidence suggested higher prevalence of dementia in Malay and Indian ethnic groups compared to the Chinese ethnic group. Even though not all studies in these comparisons were eligible for meta-analysis, the results from the narrative synthesis support those from meta-analyses.

Our results suggest that the people from the Black ethnic group have around 30% higher incidence of dementia and people from the Asian ethnic group have nearly the same incidence rate compared to people from the White ethnic group. These findings are similar to those from an earlier systematic review by Mehta and Yeo [10]. A further recent study conducted among US Veterans and published after the time period of our search supports our findings [34]. We also showed a similar increase in dementia incidence among those of Black ethnicity in a post-stroke population Shiekh et al. [11]. In contrast, Shadlen et al. [35] and Evans et al. [36] had reported no difference in incidence of dementia comparing Black and White ethnic groups, although these were not included in our review as they had different main exposures of interest. Our results show lower incidence of dementia in Latinos compared the White ethnic group than Mehta and Yeo [10]. In their systematic reviews, Chin et al. [37] suggested that the Black ethnic group has around two times the prevalence of dementia compared to White ethnic group, while Adelman et al. [38] suggested differences in risk of cognitive impairment or dementia due to cardiovascular risk factors such as undiagnosed or untreated hypertension and diabetes which are more common in ethnic minority groups. The 2020 report of the Lancet Commission on dementia prevention, intervention, and care [4] highlights the need to tackle the higher risk of dementia in ethnic minority groups in its recommendations.

Strengths of our review included that we conducted as comprehensive a search as possible. We aimed to observe differences in at least age-adjusted incidence and prevalence measured within the same study. We only included studies which are population based, mitigating selection bias. Studies conducted on institutionalized populations such as those living in nursing homes only were not included. We only included studies which compared dementia incidence or prevalence of at least two ethnicities within the same study to avoid inappropriate comparisons across studies of population level ethnic differences in dementia incidence and prevalence. Of these 10 studies were cohort studies with information on temporality to allow comparisons of dementia incidence. We also included only studies reporting age-adjusted or age-specific incidence or prevalence rates as age is an important driver of cognitive performance. Unlike many other studies of dementia risk factors, reverse causation was not a particular concern in our study as ethnicity precedes the outcome.

However, our review had some limitations which should be considered while drawing interpretations. Four of our largest studies included are based on electronic health records results from which might be affected by factors such as ethnic differences in healthcare service utilization [37]. This might be especially relevant in health insurance-based settings, such as the US. These could also be restricted in their measurement of the ethnic groupings and covariates assessed in these studies, potentially leading to biased results and residual confounding. There could be some heterogeneity in our results due to the broad time interval of included studies. We did not consider this a major limitation as comparisons were within studies. Most of the studies included in this study were recent, and among the earlier studies, only Demirovic et al. [31] reported much higher estimates than other studies in their comparison. Most of the studies included in the review looked at broad ethnic group categories, e.g., Asian and White. It is unclear whether these broad ethnic categories were developed using similar definitions. There could also be important differences in dementia incidence and prevalence concealed within these broad categories [10, 39]. Studies included varied in how they defined the ethnic categories and outcomes, with ascertainment of dementia depending on the cognitive tests or algorithms used. These could have resulted in differential misclassification if the validity of instruments varied across ethnic groups [39, 40]. Only about half of the included studies adjusted for cardiovascular risk factors for dementia such as hypertension and diabetes, or other factors such as education and socioeconomic status. There could be ethnic differences in these which could affect the results obtained.

Not all our studies reported their results with the White ethnic group as the reference. This limited the number of comparisons we could make. We calculated the effect estimates for some of these but could not generate the corresponding confidence intervals due to information on covariances not being available. Hence, we were not able to determine if these measures were only due to chance. These studies were from different time periods and locations, which might lead to differing definitions of ethnicity and dementia, in addition to real changes in population figures. Due to limited number of studies available for the comparisons made we used these when these were methodologically similar. If a greater number of studies had been available, we would have segregated our analysis into different time periods and locations to get a more accurate picture. Although we conducted our searches in seven databases, the results were dependent on articles which were indexed to be identified in English searches. Future systematic reviews could include searches in other languages.

Our study suggests important ethnic differences in dementia incidence and prevalence. Further high-quality research studies from a range of global settings are needed with sufficiently large sample sizes to enable comparisons within, as well as between, ethnic groups [39] including comparisons of more granular ethnic sub-groups which comprise the standard high-level categories of White, South Asian, and Black. Future studies might also look at secular trends in ethnic inequalities in dementia incidence and prevalence over time. It is also important to understand when and how these differences arise. Recent research highlights the clustering of risk factors among minority ethnic groups [4]. Further population based studies are required with accurately measured ethnicity, dementia, and covariate information [10, 12] to understand whether any of these differences are biological in nature and what proportion are due to differences in patterns of risk factors such as hypertension, educational levels, and socio-economic conditions over the life course. In the UK, two recently published studies show ethnic disparities in memory clinic access [41] and in psychotropic drug prescribing among patients with dementia [6]. Better understanding where ethnic differences arise in the healthcare pathway, e.g., in access to diagnosis, treatments, and end of life care, is essential to reducing inequalities. This will be helped by improved representation of people from ethnic minority groups in dementia research and trials [42, 43].

## Supplementary Material

Supplementary Material
