# Supplementary material for: Ethnic Differences in Dementia Risk: A Systematic Review and Meta-Analysis
Source: J Alzheimers Dis. 2021 Mar 9;80(1):337–55. doi: 10.3233/JAD-201209 (PMC8075390; doi:10.3233/JAD-201209)
Supplement: Supplementary Material [file jad-80-jad201209-s001.pdf]

Supplementary Material

Ethnic Differences in Dementia Risk: A Systematic Review and Meta-Analysis

Supplementary Material 1. Database searches

Database: MEDLINE
----
1 exp incidence/
2 incidence.mp.
3 exp prevalence/
4 prevalence.mp.
5 exp dementia/
6 (dementia or alzheimer* or kluver-bucy or huntington*).mp.
7 ((memory or cognit* or mental) adj5 (los* or impair* or deficit or problem or damage or declin* or deteriorate* or degenerate* or diminish*)).mp.
8 (lew? adj3 bod*).mp.
9 (((frontotemporal or fronto-temporal) and (lobar or lobe)) adj3 degeneration).mp.
10 Supranuclear Palsy, Progressive/
11 (supra?nuclear pals$ or supra-nuclear pals$ or PSP or Steele-Richardson-Olszewski syndrome or SRO).mp.
12 exp prion diseases/
13 (Prion$ disease$ or fatal familial insomnia or FFI or Gertsmann-Straussler-Scheinker syndrome or GSS or kuru or variab$ protease-sensitive prionopathy or VPSPr or transmissible spongiform encephalopath$ or TSE or Creutzfeld-Jacob$ or JCD or CJD).mp.
14 exp ethnic groups/
15 Minority Groups/
16 exp continental population groups/
17 (african* or asian* or arab* or hispanic*).mp.
18 (ethnic* or race* or racial).mp.
19 (latin* or black or white or caucasian).mp.
20 (bame or bme or minority* or caribbean or migrant* or immigrant*).mp.
21 1 or 2 or 3 or 4
22 5 or 6 or 7 or 8 or 9 or 10 or 11 or 12 or 13
23 14 or 15 or 16 or 17 or 18 or 19 or 20
24 21 and 22 and 23

Database: Embase

----

1 exp incidence/
2 incidence.mp.
3 exp prevalence/
4 prevalence.mp.
5 exp dementia/
6 (dementia or alzheimer* or kluver-bucy or huntington*).mp.
7 ((memory or cognit* or mental) adj5 (los* or impair* or deficit or problem or damage or declin* or deteriorate* or degenerate* or diminish*)).mp.
8 (lew? adj3 bod*).mp.
9 (((frontotemporal or fronto-temporal) and (lobar or lobe)) adj3 degeneration).mp.
10 progressive supranuclear palsy/
11 (supra?nuclear pals$ or supra-nuclear pals$ or PSP or Steele-Richardson-Olszewski syndrome or SRO).mp.
12 exp prion disease/
13 (Prion$ disease$ or fatal familial insomnia or FFI or Gertsmann-Straussler-Scheinker syndrome or GSS or kuru or variab$ protease-sensitive prionopathy or VPSPr or transmissible spongiform encephalopath$ or TSE or Creutzfeld-Jacob$ or JCD or CJD).mp.
14 exp ethnic group/ or exp ethnic difference/
15 Minority Groups/
16 exp ancestry group/
17 (african* or asian* or arab* or hispanic*).mp.
18 (ethnic* or race* or racial).mp.
19 (latin* or black or white or caucasian).mp.
20 (bame or bme or minority* or caribbean or migrant* or immigrant*).mp.
21 1 or 2 or 3 or 4
22 5 or 6 or 7 or 8 or 9 or 10 or 11 or 12 or 13
23 14 or 15 or 16 or 17 or 18 or 19 or 20
24 21 and 22 and 23

Database: PsycINFO

----

1 incidence.mp.
2 prevalence.mp.
3 exp dementia/
4 (dementia or alzheimer* or kluver-bucy or huntington*).mp.
5 ((memory or cognit* or mental) adj5 (los* or impair* or deficit or problem or damage or declin* or deteriorate* or degenerate* or diminish*)).mp.
6 (lew? adj3 bod*).mp.
7 (((frontotemporal or fronto-temporal) and (lobar or lobe)) adj3 degeneration).mp.
8 Progressive Supranuclear Palsy/
9 (supra?nuclear pals$ or supra-nuclear pals$ or PSP or Steele-Richardson-Olszewski syndrome or SRO).mp.
10 exp Alzheimer's Disease/ or exp Creutzfeldt Jakob Syndrome/ or exp Encephalopathies/ or exp Prion/ or exp Neurodegenerative Diseases/ or exp Nervous System Disorders/
11 (Prion$ disease$ or fatal familial insomnia or FFI or Gertsmann-Straussler-Scheinker syndrome or GSS or kuru or variab$ protease-sensitive prionopathy or VPSPr or transmissible spongiform encephalopath$ or TSE or Creutzfeld-Jacob$ or JCD or CJD).mp.
12 exp "Racial and Ethnic Differences"/ or exp "Racial and Ethnic Attitudes"/ or exp "Racial and Ethnic Groups"/
13 exp Minority Groups/
14 exp Blacks/
15 exp Southeast Asian Cultural Groups/ or exp South Asian Cultural Groups/
16 exp Arabs/
17 exp "Latinos/Latinas"/
18 exp Whites/
19 (african* or asian* or arab* or hispanic*).mp.
20 (ethnic* or race* or racial).mp.
21 (latin* or black or white or caucasian).mp.
22 (bame or bme or minority* or caribbean or migrant* or immigrant*).mp.
23 1 or 2
24 3 or 4 or 5 or 6 or 7 or 8 or 9 or 10 or 11
25 12 or 13 or 14 or 19 or 20 or 21 or 22
26 23 and 24 and 25

Database: CINAHL

----

1 (MH "Incidence")
2 incidence
3 (MH "Prevalence")
4 prevalence
5 (MH "Dementia+") OR (MH "Frontotemporal Dementia+") OR (MH "Delirium, Dementia, Amnestic, Cognitive Disorders+") OR (MH "Dementia, Multi-Infarct") OR (MH "Lewy Body Disease") OR (MH "Dementia Patients")
6 dementia OR alzheimer* OR "kluver-bucy" OR "kluver bucy" OR huntington*
7 ((memory OR cognit* OR mental) N5 (los* OR impair* OR deficit OR problem OR damage OR declin* OR deteriorate* OR degenerate* OR diminish*))
8 lew? N3 bod*
9 (((frontotemporal OR "frontotemporal") AND (lobar OR lobe)) N3 degeneration)
10 (MH "Supranuclear Palsy, Progressive")
11 ("supra?nuclear pals*" OR "supra-nuclear pals*" OR PSP OR "Steele-Richardson-Olszewski syndrome" OR SRO)
12 (MH "Prion Diseases+")
13 "Prion* disease*" OR "fatal familial insomnia" OR FFI OR "Gertsmann-Straussler-Scheinker syndrome" OR "Gertsmann Straussler-Scheinker syndrome" OR GSS OR kuru OR "variab* proteasesensitive prionopathy" OR "variab* protease sensitive prionopathy" OR VPSPr OR "transmissible spongiform encephalopath*" OR TSE OR "Creutzfeld-Jacob*" OR "Creutzfeld Jacob*" OR JCD OR CJD)
14 (MH "Ethnic Groups+")
15 (MH "Minority Groups")
16 african* OR asian* OR arab* OR hispanic*
17 ethnic* OR race* OR racial
18 latin* OR black OR white OR caucasian
19 bame OR bme OR minority* OR caribbean OR migrant* OR immigrant*
20 S1 OR S2 OR S3 OR S4
21 S5 OR S6 OR S7 OR S8 OR S9 OR S10 OR S11 OR S12 OR S13
22 S14 OR S15 OR S16 OR S17 OR S18 OR S19
23 S20 AND S21 AND S22

Database: Global Health

----

1 exp familial incidence/ or exp incidence/
2 incidence.mp.
3 "disease prevalence AND/OR seroprevalence"/
4 prevalence.mp.
5 exp dementia/
6 (dementia or alzheimer* or kluver-bucy or huntington*).mp.
7 ((memory or cognit* or mental) adj5 (los* or impair* or deficit or problem or damage or declin* or deteriorate* or degenerate* or diminish*)).mp.
8 (lew? adj3 bod*).mp.
9 (((frontotemporal or fronto-temporal) and (lobar or lobe)) adj3 degeneration).mp.
10 Alzheimer's disease.sh.
11 (supra?nuclear pals$ or supra-nuclear pals$ or PSP or Steele-Richardson-Olszewski syndrome or SRO).mp.
12 exp prion diseases/
13 (Prion$ disease$ or fatal familial insomnia or FFI or Gertsmann-Straussler-Scheinker syndrome or GSS or kuru or variab$ protease-sensitive prionopathy or VPSPr or transmissible spongiform encephalopath$ or TSE or Creutzfeld-Jacob$ or JCD or CJD).mp.
14 exp ethnic groups/
15 exp minorities/
16 (african* or asian* or arab* or hispanic*).mp.
17 (ethnic* or race* or racial).mp.
18 (latin* or black or white or caucasian).mp.
19 (bame or bme or minority* or caribbean or migrant* or immigrant*).mp.
20 1 or 2 or 3 or 4
21 5 or 6 or 7 or 8 or 9 or 10 or 11 or 12 or 13
22 14 or 15 or 16 or 17 or 18 or 19
23 20 and 21 and 22

Database: Scopus

((TITLE-ABS-KEY (incidence)) OR (TITLE-ABS-KEY (prevalence))) AND

((TITLE-ABS-KEY (dementia OR alzheimer* OR "kluver-bucy" OR huntington*)) OR (TITLE-ABS-KEY ((memory OR cognit* OR mental) W/5 (los* OR impair* OR deficit OR problem OR damage OR declin* OR deteriorate* OR degenerate* OR diminish*))) OR (TITLE-ABS-KEY ((lew? W/3 bod*))) OR (((TITLE-ABS-KEY ("fronto-temporal") AND TITLE-ABS-KEY ("(lobar OR lobe)"))) AND (TITLE-ABS-KEY (degeneration))) OR (TITLE-ABS-KEY ("supra?nuclear pals*" OR "supra-nuclear pals*" OR psp OR "Steele-Richardson-Olszewski syndrome" OR sro)) OR (TITLE-ABS-KEY ("Prion* disease*" OR "fatal familial insomnia" OR ffi OR "Gertsmann-Straussler-Scheinker syndrome" OR gss OR kuru OR "variab* protease-sensitive prionopathy" OR vpspr OR "transmissible spongiform encephalopath*" OR tse OR "Creutzfeld-Jacob*" OR jcd OR cjd))) AND

((TITLE-ABS-KEY (african* OR asian* OR arab* OR hispanic*)) OR (TITLE-ABS-KEY (ethnic* OR race* OR racial)) OR (TITLE-ABS-KEY (latin* OR black OR white OR caucasian)) OR (TITLE-ABS-KEY (bame OR bme OR minority* OR caribbean OR migrant* OR immigrant*))) AND

(LIMIT-TO (DOCTYPE , "ar"))

Database: Web of Science

----

1 TOPIC: (incidence)
2 TOPIC: (prevalence)
3 TOPIC: (dementia OR alzheimer* OR "kluver-bucy" OR huntington*)
4 TOPIC: ((memory OR cognit* OR mental) NEAR/5 (los* OR impair* OR deficit OR problem OR damage OR declin* OR deteriorate* OR degenerate* OR diminish*))
5 TOPIC: ((lew? NEAR/3 bod*))
6 TOPIC: ("fronto*temporal") AND TOPIC: (lobar OR lobe) AND TOPIC: (degeneration)
7 TOPIC: (("supranuclear pals*" OR "supra-nuclear pals*" OR PSP OR "Steele-Richardson-Olszewski syndrome" OR SRO))
8 TOPIC: (("Prion* disease*" OR "fatal familial insomnia" OR FFI OR "Gertsmann-Straussler-Scheinker syndrome" OR GSS OR kuru OR "variab* protease-sensitive prionopathy" OR VPSPr OR "transmissible spongiform encephalopath*" OR TSE OR "Creutzfeld-Jacob*" OR JCD OR CJD))
9 TOPIC: ((african* OR asian* OR arab* OR hispanic*))
10 TOPIC: ((ethnic* OR race* OR racial))
11 TOPIC: ((latin* OR black OR white OR caucasian))
12 TOPIC: ((bame OR bme OR minority* OR caribbean OR migrant* OR immigrant*))
13 #2 OR #1
14 #8 OR #7 OR #6 OR #5 OR #4 OR #3
15 #12 OR #11 OR #10 OR #9
16 (#15 AND #14 AND #13) AND DOCUMENT TYPES: (Article OR Abstract of Published Item)
